# Bactericidal activities of GM flax seedcake extract on pathogenic bacteria clinical strains

**DOI:** 10.1186/1472-6750-14-70

**Published:** 2014-07-29

**Authors:** Magdalena Zuk, Agata Dorotkiewicz-Jach, Zuzanna Drulis-Kawa, Malgorzata Arendt, Anna Kulma, Jan Szopa

**Affiliations:** 1 Faculty of Biotechnology, Wrocław University, Przybyszewskiego 63/77, 51-148 Wroclaw, Poland; 2 Institute of Genetics and Microbiology, Wroclaw University, Przybyszewskiego 63/77, 51-148 Wroclaw, Poland; 3 Linum Fundation, Stabłowicka 147/149, 54-066 Wrocław, Poland

**Keywords:** Antimicrobial compound, Phenolic acid, Flax, Alternative antibiotic, Flax seedcake

## Abstract

**Background:**

The antibiotic resistance of pathogenic microorganisms is a worldwide problem. Each year several million people across the world acquire infections with bacteria that are antibiotic-resistant, which is costly in terms of human health. New antibiotics are extremely needed to overcome the current resistance problem.

**Results:**

Transgenic flax plants overproducing compounds from phenylpropanoid pathway accumulate phenolic derivatives of potential antioxidative, and thus, antimicrobial activity. Alkali hydrolyzed seedcake extract containing coumaric acid, ferulic acid, caffeic acid, and lignan in high quantities was used as an assayed against pathogenic bacteria (commonly used model organisms and clinical strains). It was shown that the extract components had antibacterial activity, which might be useful as a prophylactic against bacterial infection. Bacteria topoisomerase II (gyrase) inhibition and genomic DNA disintegration are suggested to be the main reason for rendering antibacterial action.

**Conclusions:**

The data obtained strongly suggest that the seedcake extract preparation is a suitable candidate for antimicrobial action with a broad spectrum and partial selectivity. Such preparation can be applied in cases where there is a risk of multibacterial infection and excellent answer on global increase in multidrug resistance in pathogenic bacteria.

## Background

The worldwide increase in the occurrence of multidrug resistance (MDR) in bacterial pathogens has become a serious problem in the clinical treatment of infections [1]. We are witnessing a high increase in the resistance levels of pathogenic bacteria and a sharp decrease in the efficacy of conventional antibacterial therapy, often across the whole spectrum of known therapeutics [2-4]. Even chemotherapeutics that are used as the last line of defense is less effective in an eradication of infections involving MDR strains. Bacteria can develop or acquire new mechanisms of resistance and share the information by horizontal transfer between species. Nowadays, over 90% of staphylococci, pneumococci and enterococci isolated from severe infections are resistant to antibiotics. Increasing prevalence of methicillin-resistant *Staphylococcus aureus* (MRSA), β-lactam- and macrolide-resistant pneumococci, and glycopeptide- and vancomycin-resistant enterococci have been reported [5]. Among the Gram-negative bacteria, the most serious treatment problems are caused by MDR *Pseudomonas aeruginosa* strains producing carbapenemases and MDR *Enterobacteriaceae* strains including *Escherichia coli* and *Klebsiella pneumoniae*, which produce extended beta-lactamases (ESBL) [6]. In the WHO (Word Health Organisation) report from World Health Day 2011 *Antimicrobial resistance*: *no action today*, *no cure tomorrow*, it was indicated that 150,000 people die each year as a result of only one very dangerous disease, multidrug-resistant tuberculosis (MDR-TB) [7]. The global increase in multidrug resistance in pathogenic bacteria has led to an increasing need for topical antimicrobial products that can be applied in cases of multibacterial infection. Many of the available agents have highly bactericidal activity, but their cytotoxicity can also interfere with human tissues [8]. Actually one of the great challenges of modern bio-medical science is searching for compounds/agents that can be simultaneously effective as an unselective antibiotic and not indicate cytotoxic effect to human cells. Such preparations will be useful in the case of multibacterial and fungal infections.

Bioactive components isolated from plants are one alternative to commercially available traditional antibiotic. The rich source of bioactive metabolites may be flax (*Linum usitatissimum* L.), an annual plant that grows widely in the Mediterranean and temperate climate zones. It is not only a source of oil and fibers but also seedcake, the remains of the flax seeds after oil extraction. Seedcake has important dietary properties, and it is also used against many diseases, such as skin, respiratory tract and gastrointestinal tract diseases [9-11].

The health-promoting properties of flax can be further improved by genetic modification.

Findings presented in this paper indicated extensive spectrum antibacterial activity of preparation based on flax seedcake. The material for that set of experiments was derived from transgenic flax plants designated W92. They were obtained by plant transformation using the genes coding chalcone synthase (*CHS*), chalcone isomerase (*CHI*) and dihydroflavonol reductase (*DFR*), which all control the synthesis of antioxidative compounds generated via the phenylpropanoid pathway [12].

Significant increases in the amounts of various flavonoids (kaempferol, quercetin) were reported in the seeds of transgenic W92 flax. The levels of anthocyanins, proanthocyanidins and phenolic acids were also higher in the W92 flaxseeds than those of the control plants [13]. Ferulic, caffeic and p-coumaric acids and their glucoside derivatives were the most common phenolic acids present. Three main compounds from the phenylpropanoid pathway were identified in the fibers from W92 plants: vanillin, 4-hydrobenzoic acid and acetovanillone. UPLC analysis of fibers from W92 flax also revealed the presence of ferulic acid. The same compounds but in significantly lower amounts and different proportion between individual metabolites will be found in unmodified plants. Therefore, for further experiments on clinic bacterial stains, we used seedcake from W92 transgenic flax.

Detailed analysis of seedcake extract from transgenic flax indicates that such preparation is effective as an antibiotic wide spectrum of bactericidal activity, useful in the case of multibacterial and fungal infections is the main goal of this study. The data obtained strongly suggest that the seedcake extract a suitable candidate for antimicrobial action with a broad spectrum and partial selectivity. It is believed that this is the first report describing the potential of a product from GM flax for antimicrobial application.

## Methods

### Plant material

All of the presented experiments were performed on flax plants (*Linum usitatissimum L*.). Seeds from unmodified flax (cv. *Linola*) were obtained from the Flax and Hemp Collection of the Institute of Natural Fibers, Poland. Transgenic plants overproducing phenylpropanoid compounds were previously generated in laboratories of the University of Wroclaw [12] In brief, to construct the transgenic plants, we used a binary vector containing three cDNAs from *Petunia hybrida* encoding chalcone synthase [CHS, EMBL/GenBank database acc. no. *X04080*], chalcone isomerase [CHI, EMBL/GenBank database acc. no. *X14589*] and dihydroflavonol reductase [DFR, EMBL/GenBank database acc. no. *X15537*] in the sense orientation, under the control of the 35S promoter and OCS terminator. The vector was introduced into *Agrobacterium tumefaciens* and used for flax (cv. *Linola*) transformation. The transgenic plants were preselected via PCR using primers specific for the kanamycin resistance gene (*npt II*). The relevant selection of transgenic plants was performed by Northern blot analysis using radiolabeled cDNAs of all three transgenes (CHS, CHI, and DFR) as probes. The details on plant transformation, selection and transgenic plant analysis were described previously [12,14]. The reference specimens were collected in the University of Wroclaw Biotechnology Department in the form of tissue culture plants and seeds from all of the cultivation seasons.

In this study, we used the fifth generation transgenic plants W92 (transgenic line W92.40) and unmodified flax plants cv. Linola (as a control), both were field-grown in the 2011 growing season. The 600 m2 was seeded (sow density 600seeds/m2) and 12 kg of seeds, 15 kg stalks and finally 4,5 kg fiber for W92 as well as control plants were obtained. The seeds were harvested 4 months after sowing and used for oil pressing. The stems of plants were then retted by the dew method as described previously [15]. Briefly, the harvested plant stalks were spread evenly in field for at least 40 days. Combined action of water (dew), sun and naturally occurring in a soil bacteria and fungi caused degradation of the cell-wall polysaccharides and middle lamella, releasing the fibers from the stems. The retted stalks, called straw, were dried in open air and were stored for a short period (one week) to allow curing to occur, facilitating fibre removal. Final separation of the fibre is accomplished by a breaking process in which the brittle woody portion of the straw is broken, which removes the broken woody pieces (shaves) by beating or scraping.

### Seedcake preparation

Flaxseeds (10 kg) were ground and transferred to an industrial warm gear oil press (Oil PressDD85G – IBG Monoforts Oekotec GmbH& Co) to be cold pressed at a maximum of 40°C. After the cold pressing, 5 to 7% of the total fatty acids that are present in untreated seeds normally remain in the seedcakes as a residual oil fraction. In order to remove all of the fat from the seedcakes, the ground material (~100 μm grain) was defatted by threefold extraction with hot (65°C) hexane, after such treatment obtain 7.6 kg fully defatted seedcakes. Afterward, the seedcakes were dried and used for further analysis and for the preparation of the alkali hydrolyzed seedcake extract.

### Determination of total phenolic content

The plant material (seeds, seedcakes and fibers) was crushed using a laboratory mill. The seeds and seedcakes were defatted using hot hexane before analysis. A 250-mg sample of each material was extracted three times with 800 μl of methanol containing 1% formic acid, and sonicated for 15 min. Next, the samples were centrifuged (10 min at 14000 g) and the clear supernatant was used for the analysis. The total phenolics content was determined by the optimized Folin-Ciocalteau method [16], referring to the calibration curve of gallic acid, which is a phenol compound used as a standard. A total of 100 μl of each sample solution was mixed with 0.2 ml of Folin-Ciocalteau reagent, 2 ml of H_2_O, and 1 ml of 15% Na_2_CO_3_. The absorbance was measured with a model spectrophotometer at 765 nm after 2 hours incubation at room temperature. The total phenolics were estimated as gallic acid equivalent. The data were obtained from the average of three determinations.

Determination of total anthocyanin content via the pH-differential method [17] Samples of the ground and defatted seeds, seedcakes (15 mg) and fibers (15 mg) were extracted with 1 ml of methanol/HCl (95:5, v/v) in an ultrasonic bath for 30 min. The extract was centrifuged at 14000 g for 10 min. Two dilutions of the sample were performed: first, 100 μl of the supernatant was mixed with 900 μl of 0.025 M potassium chloride buffer, pH 1.0, and then 100 μl of supernatant was mixed with 900 μl of 0.4 M sodium acetate buffer, pH 4.5. The solution was allowed to stand at room temperature for 15 min, and then the absorbance at 520 nm and 700 nm was measured, which allowed for haze correction. The results were reported as cyanidin-3-*O*-glucoside equivalents.

### Evaluation of proanthocyanin content

For the measurements, 15 mg samples of defatted seeds and seedcakes were used. Proanthocyanins were hydrolyzed with 1 ml of n-butanol/HCl (95:5, v/v) and 33 μl of 2% (w/v) NH_4_Fe(SO_4_)_2_ 12H_2_O in 2 M HCl for 40 min at 95°C. The extract was centrifuged at 14000 g for 10 min, and the supernatant was used for proanthocyanin content evaluation. Proanthocyanin detection was carried out by measuring absorption at 540 nm, and proanthocyanin content was expressed as catechin equivalents [18].

### Measurement of phenolic acid and secoisolariciresinol diglucoside (SDG) contents

A 250 mg sample of defatted flax seeds or seedcakes was extracted three times with 1.5 ml of 80% methanol (v/v) for 10 min at 80°C. Prior to extraction, the seedcakes were finally defatted, again with hot hexane. The extracts were centrifuged and evaporated to near dryness at 40°C under a vacuum. The extract was then resuspended and subjected to alkaline hydrolysis (1 ml, 0.3 M aqueous sodium hydroxide) for 2 days at room temperature followed by neutralization using 2 M HCl to pH = 6.0. The extract (3× 1-3 μl injection) was analyzed on a Waters Acquity UPLC (Ultra Performance Liquid Chromatography) system with a 2996 PDA detector, using Acquity UPLC column BEH C18, 2.1100 mm, 1.7 μm. The mobile phase was A = acetonitrile and B = 20 mM ammonium formate, pH 3.0 in a gradient flow: 1 min, 10% A/90% B; 2–6 min gradient to 40% A/60% B, and 7 min gradient from 40% to100% A with a 0.4 ml/min flow rate [19]. Measurements were taken at 280 (SDG) and 320 nm (phenolic acids).

### Extraction of phenylpropanoids from fibers

One gram of W92 flax fibers was ground in a Retch mill to a fine powder (100 μm grain) and extracted three times with10ml methanol. Extracts were pooled, evaporated under a vacuum and resuspended in 2 ml methanol. The remaining matter was hydrolyzed in 2 N NaOH at room temperature for 24 hours in order to release bound phenolics. Extracts were adjusted to pH 3.0 extracted three times with ethyl acetate, pH3.0 and then the extract was evaporated under a vacuum and resuspended in 2 ml of methanol.

### UPLC analysis of phenolics in flax fibers

The components were analyzed using the Acquity UPLC system (Waters) equipped with an automated sample injector and PDA detector. A 10-μl sample was applied to an Acquity UPLC HSS- T3 column, 2.1 × 100 mm, 1.8 μm retaining better hydrophilic components. The mobile phase was passed through the column at a flow rate of 0.5 ml/min. The mobile phase consisted of 0.1% formic acid (A) and 100% methanol (B). For the first 2 min, isocratic elution was carried out using 100% of A, 2–5 min a linear gradient to 30% A/70% B, 5–5.5 min to 0% A/100% n B. In the final minute (5,5-6,5 min) the concentration of A was returned to 100%. An additional analysis of the very hydrophilic component was performed using a UPLC HILIC, 2.1 × 100 mm, 1.7 μm column. The mobile phase was passed through the column at a flow rate of 0.4 ml/min. The mobile phase consisted of 0.1% formic acid (A) and 100% acetonitrile (B). For the first 4 min 10% A/90% B was used, 4–8 min a linear gradient to 90% A/10% B. was applied, 8 - 9 min the gradient to 100% A was used. In the final minute, the concentration of eluting solvents was returned to 10% A /90% B [13].

### Preparation of alkali hydrolyzed seedcake extract

Preparation was performed according modified method previously described by Zuk et al. [13] Defatted flax seedcakes (100 g) were extracted three times with 400 ml of 80% methanol (v/v) for 15 min at 80°C. The extract was centrifuged, the pellet was discarded and methanol from the supernatant fraction was evaporated at 40°C. The aqueous fraction of the extract was subjected to alkaline hydrolysis in a final concentration of 0.3 M sodium hydroxide for 2 days at room temperature. The hydrolyzed syrup was acidified with 2 M hydrochloric acid to pH 6.0.The solution was cooled down to 10°C then centrifuged with a high-speed centrifuge at 7000 rpm for 15 min to precipitate and remove water-soluble polysaccharides and proteins. After freeze-drying, the dry material was preserved at 4°C and used to prepare active water solutions (w/v) of 50, 30 and10mg/ml. The solutions were sterilized by filtration through an Acrodisc 0.22 μm filter (Gelman Sciences, Ann Arbor, MI).

### Antioxidant capacity measurement *via* chemiluminescence method

The different seedcake extract solutions (from control and GM plants) or active seedcake preparation at a concentration of 9.5 mg/ml, which is the equivalent of 10 mM gallic acid in the total phenolic measurement, and a 10-mM solution of standard substances (ascorbic acid, chlorogenic acid, caffeic acid, SDG) were diluted in the range of 1000–15000 times with water, and directly analyzed according to the published method [20].This experiment was performed in a final volume of 250 μl on white microplates in a solution containing 0.1 M Tris–HCl buffer, pH 9.0, and 4 mM 2,2-azobis (2-amidinopropane) dihydrochloride (AAPH), freshly prepared. The luminol solution (100 mM) and diluted extract were automatically injected. The photons produced in the reaction were counted on an EG&G Berthold LB96P microplate luminometer at 30°C. The antioxidant potential (IC50) was defined as the amount of flax extract (mg FW) that inhibits luminol chemiluminescence by 50%.

### Bacterial strains

Thirty-six Gram-negative clinical strains (*Pseudomonas aeruginosa* [n = 16], *Klebsiella pneumoniae* [n = 10] and *Escherichia coli* [n = 10]) and 15 Gram-positive clinical strains (*Staphylococcus aureus* [n = 5], *Staphylococcus epidermidis* [n = 5] and *Enterococcus faecalis* [n = 5]) were used to determine the antibacterial activity of seedcake extract. The tested strains were isolated from clinical samples from patients hospitalized in the Lower Silesian Centre of Pediatrics in Wroclaw, Poland. MDR isolates were included in the selection (Additional file 1: Table S1 and Table S2). As references, we used the strains *P. aeruginosa* ATCC 27853, *K.pneumoniae* ATCC 700603, *E. coli* ATCC 25922, *E.coli* ATCC 11229, *S. aureus* ATCC 29213 and *S. aureus* ATCC 6538 from the American Type Culture Collection (LGC Standards, Lomianki, Poland). The bacteria were stored at -70°C in Trypticase Soy Broth (Becton Dickinson and Company, Cockeysville, MD, USA) supplemented with 20% glycerol.

### Determination of bacterial susceptibility

The antimicrobial activity of the seedcake extract was measured by determining the minimum inhibitory concentration (MIC). The MIC tests were performed by a broth microdilution method according to the European Committee on Antimicrobial Susceptibility Testing standards (EUCAST, http://www.eucast.org). Three different dilutions of W92 flax seedcake extract were prepared: 50, 30 and 10 mg/ml (for details see Table 1). The proper concentration of seedcake extract was prepared by mixing with Mueller Hinton II Broth powder (MHB; Becton Dickinson and Company, Cockeysville, USA) and sterilized by filtration. The gentamicin (GM) and ampicillin (AM) for antibiotic susceptibility tests were obtained from Sigma-Aldrich Chemie GmbH (Steinheim, Germany). The serial concentration of antibiotics were prepared in MHB according to EUCAST standards [21]. For the experiments, bacterial strains were inoculated onto blood agar plates, incubated for 18 hours at 35°C, dissolved in PBS to an optical density equal to McFarland No. 0.5 and then diluted 1:10 (to approximate concentration 10^7^ cells/ml). 10 μl samples of bacterial culture were added to a 96 microtitre plate containing 200 μl of the extract solution in MHB/ antibiotic solution in MHB. The final concentration of microorganisms was 5×10^5^cfu/ml. The plates were then incubated for 18 hours at 35°C. The MIC was defined as the lowest concentration of seedcake extract at which no visible growth of bacteria was observed after 18 hours incubation. Positive controls (growth) consisted of bacteria in broth. Negative controls (sterility) consisted of uninoculated broth with which each of the extract dilutions was performed. Each assay was performed in triplicate and repeated three times on a different day to ensure reproducibility of the results.

**Table 1:** **Content of phenylpropanoid compounds in three preparations of W92 seedcake extract** (**10 mg**/**ml**,**30 mg**/**ml and 50 mg**/**ml**) **dissolved in water**

**Compounds**	**Compounds content in dried extract (mg/gDW)**	**Preparations compounds content in:**		
		**50 mgDW/ml**	**30 mgDW/ml**	**10 mgDW/ml**
Secoisolariciresinol diglucoside (SDG)	548.41 ± 9.56	27.4 ± 0.03	16.0 ± 0.02	5.5 ± 0.02
Ferulic acid	16.09 ± 0.63	0.803 ± 0.00	0.459 ± 0.04	0.178 ± 0.00
Ferulic acid glucoside	29.55 ± 0.31	1.476 ± 0.01	0.866 ± 0.10	0.302 ± 0.02
*p*-coumaric acid	4.94 ± 0.12	0.254 ± 0.01	0.143 ± 0.02	0.049 ± 0.00
Coumaric acid glucoside	23.83 ± 0.62	1.185 ± 0.00	0.743 ± 0.04	0.238 ± 0.01
Caffeic acid	0.32 ± 0.001	0.016 ± 0.00	0.008 ± 0.00	0.003 ± 0.00
Caffeic acid glucoside	38.18 ± 0.51	1.883 ± 0.02	1.093 ± 0.02	0.378 ± 0.01

The results are the mean values ± SD (n = 6).

### Dynamics of microbial growth in the presence of seedcake extract

*In vitro* killing curves were determined in the presence of 10, 30 and 50 mg/ml seedcake extracts for the reference strains *P. aeruginosa* ATCC 27853, *K. pneumoniae* ATCC 700603, *E. coli* ATCC 11229 and *S. aureus* ATCC 6538. The control consisted of bacteria in MHB alone. The proper concentration of seedcake extract was prepared by mixing with MHB and sterilized by filtration. For the experiments, bacterial strains were inoculated onto blood agar plates, incubated for 18 hours at 35°C, dissolved in PBS to an optical density equal to McFarland No. 0.5 and then diluted 1:10 (to approximate concentration 10^7^ cells/ml). 50 μl samples of bacterial culture were added to eppendorf tube containing 1000 μl of the extract solution in MHB. Samples of 10 μl were collected after 0, 2, 4, 8 and 24 hours of incubation, serially diluted and plated on Mueller Hinton II Agar (BioMerieux, France). The bacterial count (cfu/ml) was determined after 18 hours of incubation at 35°C. Each assay was performed in triplicate.

### The inhibitory effect of standard substances on bacterial growth

For the experiments, *S. aureus* ATCC 6538 was inoculated onto blood agar plates, incubated for 18 hours at 35°C, and then diluted in PBS to an optical density equal to McFarland No. 0.5. A 10-μl sample of bacterial culture was diluted 1:10 (approximate concentration: 10^7^ cells/ml) and added to the 96 microtitre plate containing the commercially available standard substances solution (ferulic acid, coumaric acid, caffeic acid, SDG) and seedcake extract in MHB. The final concentration of microorganisms was 5×10^5^ cfu/ml. The plates were then incubated for 6 hours at 35°C. The growth inhibition effect (% of inhibition in relation to untreated bacteria) was calculated after 6hoursof incubation by measuring the OD600. Each assay was performed in triplicate.

### The inhibitory effect of seedcake extract on bacteria gyrase activity

Reaction mixture (total volume 30 μl) consisted of 5× buffer for enzyme (NEB), sterile deionized water, gyrase from *E.coli* (NEB), substrate for gyrase (NEB) and particular amount of seedcake extract (to the final concentration 10 mg/ml; 30 mg/ml; 50 mg/ml and 75 mg/ml of dried extract respectively). Covalently closed substrate (pUC ) in a relaxed DNA form was used. As negative control reaction mixture without gyrase or extract was used. As total inhibition control reaction mixture with novobiocin and without extract was used. Samples were incubated for 30 minutes at 37°C. Electrophoresis was run in 1×TBE for 2 hours in 0.8% agarose gel without ethidium bromide.

### Pulse-field gel electrophoresis of *E.coli* DNA upon seedcake extract treatment

Bacteria cells (10^8^ CFU/ml) incubated 1 hours with seedcake extract were centrifuged, pellet was resuspended in 200 μl PBS, embedded in 2% low melting agarose, then were treated first with lysozyme (10 μg/ml, 40 min at 30°C) and thereafter overnight with proteinase K (50 μg/ml at 56°C). Agarose plugs were washed 3 times with TE buffer and subjected to pulse-field electrophoresis. Gel was run in 0.5% TBE, t1 = 1 s, t2 = 50s with included angle 120°, at 12°C for 16.5 hours. After electrophoresis gel was stained with ethidium bromide and analysed under UV light (Gel Doc Imaging System, UVP Analitic).

### Skin irritation test

An *in vitro* skin irritation test was performed according to the MTT Effective Time-50 (ET-50) protocol developed at MatTek Corporation with the use of the EpiDerm skin irritation test. This test allows the valuation of skin irritation due to cosmetic ingredients and readymade products. 100 μl of three preparations of seedcake extracts (10 mg/ml, 30 mg/ml, 50 mg/ml) were put on the surface of the epidermis model. As a positive control (substance elicid skin irritation) the 1% Triton X-100 was used. After an incubation period of 2, 5 or 18 h, cell viability was assessed using the MTT colorimetric test. 50 μL of MTT stock solution (4 mg/mL) was added to each well to give a total reaction volume of 550 μL. After incubating for 4 h, the medium with MTT solution was removed from the plate. The formazan crystals in each well were dissolved in 500 μL of DMSO and incubated for 30 min with gentle shaking. The absorbance at 540 nm was read on an Asys UVM340 Microplate Reader (Biochrom, UK). The results were presented in% as a reference to the control (100%). Skin irritation potential is predicted if the remaining relative cell viability is below 50%. The experiment was performed in duplicate.

### Statistical analysis

Pearson correlation quotient was calculated. The correlations were considered significantly significant at p < 0,05. All statistical calculations were performed using STATISTICA 7.1 software package (StatSoftPolska, Poland).

## Results

Transgenic W92 flax (line W92.40) was previously obtained by simultaneous overexpression of three genes encoding key enzymes of flavonoid biosynthesis (CHS, CHI and DFR) under the control of the 35S promoter. Since the constitutive promoter was used for the modification, changes in compound contents were expected in the whole plant body. Increased amounts of many phenolic compounds (lignans, flavonoids, phenolic acids) were indeed detected in both the green parts and the seeds of W92 flax plants [13]. As a control, the level of phenolic compounds in unmodified plants was also measured.

### Biochemical analysis of seedcakes and fibers from wild type and W92 plants

First, we identified the plant product that is most suitable for the isolation of antimicrobial compounds. There are two main products that are obtained from flax: seeds, the source of oil and seedcake, which is the material remaining after oil pressing; and fiber, which is derived from the plant stem. A detailed analysis of the phenylpropanoid contents in these products from control and transgenic plants is presented in Table 2. The comparison of phenylpropanoids content firstly revealed much higher amounts of these compounds in the seedcakes than in the fiber and secondly higher level of this compounds in raw products from genetically modified plants in comparison to control.

**Table 2:** Biochemical analysis of seedcakes and fibers from wild type (WT) modified (W92) flax

**Compounds**	**WT seedcakes (mg/gFW)**	**W92 seedcakes (mg/gFW)**	**P value transgene *vs*. control**	**WT fibers (mg/gFW)**	**W92 fibers (mg/gFW)**	**P value transgene *vs*. control**
Anthocyanins	0.004 ± 0.000	0.013 ± 0.001	0.006	<0.001	<0.001	
Proanthocyanidin	0.021 ± 0.002	0.037 ± 0.003	0.02	<0.001	<0.001	
Secoisolariciresinol diglucoside (SDG)	12.112 ± 0.03	39.785 ± 0.0	0.005	0.348 ± 0.03	1.056 ± 0.05	0.01
Ferulic acid	0.57 ± 0.029	1.16 ± 0.035	0.01	0.082 ± 0.05	0,350 ± 0.08	0.002
Ferulic acid glucoside	1,488 ± 0.041	2.14 ± 0.053	0.02	0.29 ± 0.04	1.09 ± 0.05	0.002
Coumaric acid	0.340 ± 0.019	0.368 ± 0.016	0.04	<0.001	<0.001	
Coumaric acid glucoside	1.006 ± 0.030	1.721 ± 0.032	0.006	<0.001	<0.001	
Caffeic acid	0.006 ± 0.000	0.011 ± 0.001	0.06	<0.001	<0.001	
Caffeic acid glucoside	0.636 ± 0.009	1.336 ± 0.007	0.01	<0.001	<0.001	
p-Hydroxybenzoic acid	0.004 ± 0.001	0.011 ± 0.001	0.06	0,29 ± 0.01	0,350 ± 0.01	0.04
Flavonols	0.013 ± 0.001	0.022 ± 0.001	0.005	<0.001	<0.001	
Vanillin	<0.001	<0.001		0.193 ± 0.01	0.480 ± 0.01	0.02
Acetovanillone	<0.001	<0.001		0.073 ± 0.01	0.100 ± 0.003	0.01

The results are the mean values ± SD (n = 6).

The total phenolic contents in seedcakes from transgenic W92 plants (46,6 mg/gFW) is significantly higher when in control- unmodified plants (16,2 mg/gFW). Secoisolariciresinol diglucoside (SDG) is the main phenylpropanoid compound identified in seedcakes, and its content -12,1 mg/g FW for control and 39.78 mg/g FW for W92plants is respectively about75% and 85% of the total phenolic content. The UPLC analysis also revealed the presence of SDG in the fibers but a much lower quantity (1.05 mg/gFW for transgenic plants). Other compounds with antioxidant, anti-inflammatory and antibacterial properties were also analyzed. Flavonols (kaempferol and quercetin), anthocyanins and proanthocyanidins were only detected and analyzed in the seeds and seedcakes; they were not detected in the fibers. We detected phenolic acids in control and transformed flax seeds, seedcakes and fibers. The main phenolic acids in the seeds and seedcakes were ferulic acid, *p*-coumaric acid and caffeic acid and their glycoside derivatives [13]. Ferulic acid is also the most common phenolic acid in the fiber. However, the content of phenolic acids in the fibers was much lower than in the seedcakes. The compounds that were only identified in the fibers were vanillin and acetovanillone.

The general conclusion that might be drawn from the data is that seedcakes are the richest source of phenylpropanoid compounds in the plant and the level of this compounds in modified flax plants is significantly higher than in control plants. Therefore, for further experiments, we used seedcakes as a source of antimicrobial compounds.

### Preparation of seedcake extract and study of antimicrobial activities on standard (reference) pathogenic bacterial strains

The seedcake extracts from control-wild type (WT) and W92 plants were prepared (dry material) as described in Methods. The content of particular constituents was verified by UPLC analysis and proportion between active compound are similar to observed while row seedcakes analysis (data not shown). Finally, dry extracts were dissolved in water to a final concentration (w/v) of 50, 30 and 10 mg/ml. The antioxidant and antibacterial properties of such obtained preparation were analysed, in a purpose, to find the best source to generate antimicrobial preparation based on flax. All preparation were analysed to measure total phenolic content (as was expected the highest in preparation from W92 plants) and antioxidant potential (W92 plants preparation indicate almost three time better antioxidant properties – lower IC-50 parameter than unmodified plants preparation). Finally the antimicrobial activity of seedcake preparations were tested on reference (usually used for antibiotics testing) bacterial strains. The low concentration of seedcake preparation from control plants do not show any antibacterial properties, only preparation 50 mg/ml indicate such properties (see Table 3) but this activity was predominantly lower (except E. coli ) then observed for five time lower concentration of W92 based preparation. The comparable (50 mg/ml) concentration made from transgenic plant seedcakes kills all tested bacteria. As well lower (30 mg/ml) concentration of seedcake preparation based on W92 plants indicates high bactericidal activity reduction of bacterial from 87% to 100%. The obtained results to allow us to claim that seedcakes from W92 plants are the best source for antimicrobial preparation creation.

**Table 3:** **The total phenolic content**, **antioxidant potential of dried seedcake extract preparation from wild type and modified flax plants and effect on reference bacterial strains**

	**WT seedcakes dried extract**	**W92 seedcakes dried extract**		
	**50 mgDW/ml preparation**	**50 mgDW/ml preparation**	**30 mgDW/ml preparation**	**10 mg/ml preparation**
Total phenolics content [mg/ml]	24.26 ± 0.03	31.59 ± 0.05	18.45 ± 0.03	6.03 ± 0.06
IC-50 [μl]*	27.5 ± 0.04	10.56 ± 0.03	18.15 ± 0.03	26.36 ± 0.04
*K. pneumoniae* ATCC 700603**	64% ± 3.03	100% ± 0.00	98% ± 0.01	72% ± 0.93
*S. aureus* ATCC 29213**	52% ± 2.01	100% ± 0.01	93% ± 1.03	60% ± 0.63
*P. aeruginosa* ATCC 27853**	46% ± 3.50	100% ± 0.00	100% ± 0.02	85% ± 1.05
*E. coli* ATCC 25922**	75% ± 1.89	100% ± 0.23	87% ± 1.83	48% ± 1.82

The antimicrobial activity of seedcake preparations were tested on reference bacterial strains. The low concentration of seedcake preparation from control plants (10 mg/ml and 30 mg/ml) do not show any antibacterial properties (data not shown).

*The antioxidant potential (IC50) was defined as the amount (μl) of preparation that inhibits luminol chemiluminescence by 50%. **The presented results are the inhibition rate compared to the untreated bacteria (% of growth inhibition) after 6 h treatment. The results are the mean values ± SD (n = 5).

### Antioxidant properties of seedcake extract

**Figure 1:**
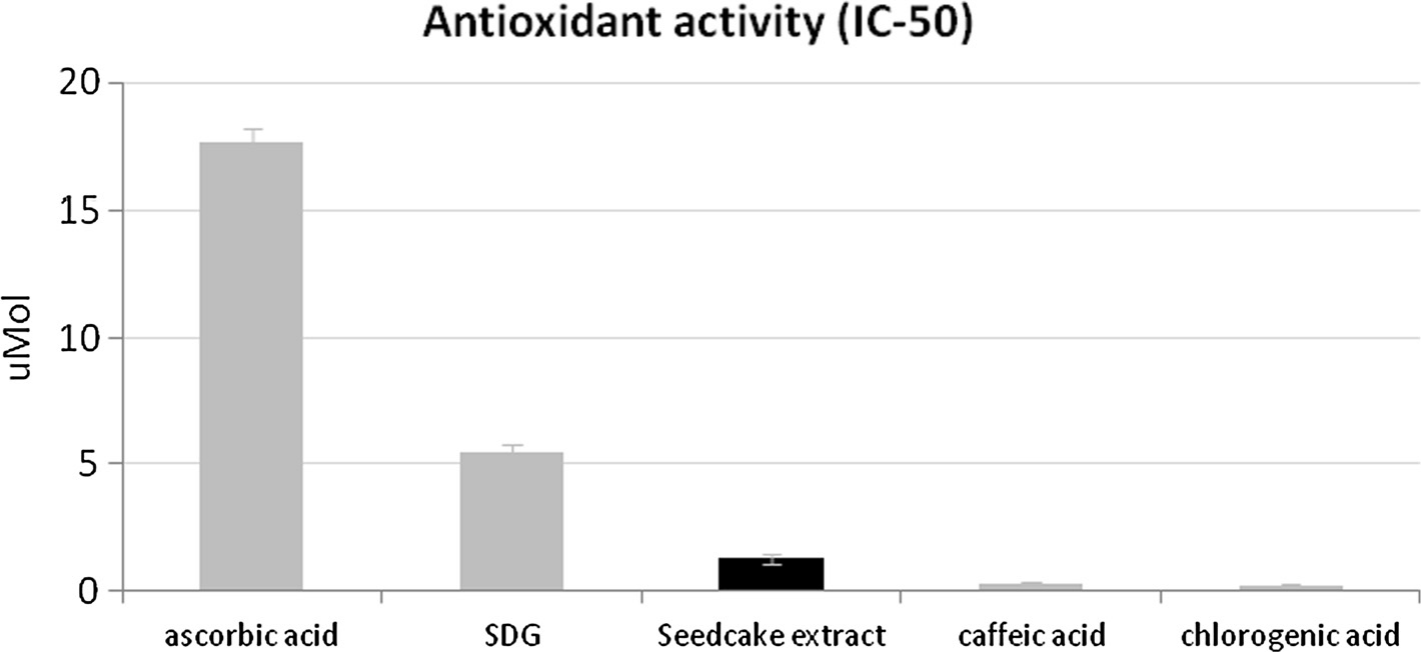
**The antioxidant activities of seedcake extract and equimolar contents of standard antioxidants.** The antioxidant activity was indicated as the IC-50 parameter. The results are the mean values ± SD (n = 6). The results are statistically significant (P < 0.05).

The process of microbial infection is usually started by oxygen free radical synthesis. Therefore, addition of external antioxidants might be helpful in arrest bacterial growth. Therefore, the antioxidant potential of the seedcake extract was measured using a chemiluminescence method, as described elsewhere [22]. The antioxidant potential was expressed as the IC_50_ value, which means the amount of extract inhibiting the oxidation of luminol by 50%. The antioxidant properties of the seedcake preparation were compared to those of well-known water-soluble antioxidants (ascorbic acid, chlorogenic acid, caffeic acid) and SDG standard solutions. The data are presented in Figure 1. It was found that the preparation made from seedcakes exhibits very high antioxidant potential: 13.5-fold higher than ascorbic acid and more than 4-fold higher than SDG. However, it was lower than that of the strong antioxidants caffeic acid (4-fold lower) and chlorogenic acid (6-fold lower). The very high antioxidative status of the preparation suggests that it may have great biomedical potential not only as an antimicrobial but also as an anti-inflammatory agent.

### Antimicrobial properties of seedcake preparations

Next, the antimicrobial properties of seedcake extract on clinical strains of pathogenic bacteria were investigated. Three different concentrations of W92 flax seedcake preparation (50, 30 and 10 mg/ml; for details see Table 1) were prepared. The levels of particular active compounds in all tested dilutions and dry extract were measured and presented in Table 1. The content of phenolic acids, SDG and other compounds are proportional to values observed while row seedcake analysis and proportion of the particular compound do not change after freeze drying and dilution process. Therefore, such method of active solution preparation obtaining procedure can be used also in the future without fears of biological activity of achieved preparations.

**Figure 2:**
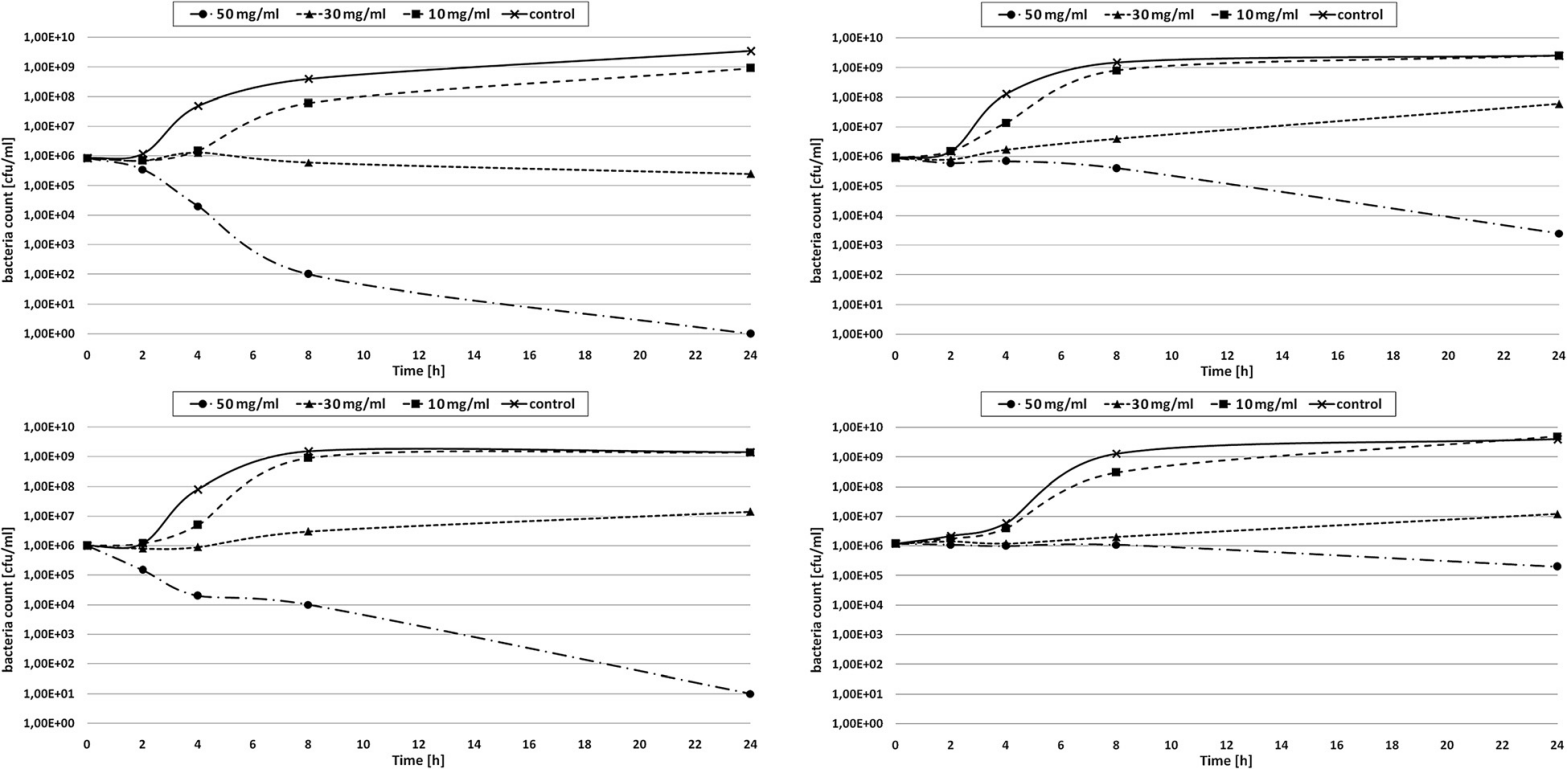
**The antibacterial activity of seedcake extract on pathogenic bacteria.** Growth curves of: *Pseudomonas aeruginosa* ATCC 27853, *Klebsiella pneumoniae* ATCC 700603, *Escherichia coli* ATCC 25922 and *Staphylococcus aureus* ATCC 29213 strains exposed to seedcake extract (preparation in a concentration of 10 mg/ml, 30 mg/ml and 50 mg/ml). The results are the mean values ± SD (n = 3). The results are statistically significant (P < 0.05).

The MIC values for control and clinical bacterial strains exposed to the seedcake preparations are presented in Tables 4 and 5. From the data obtained, it is clear that Gram-negative bacteria are more susceptible to the seedcake extract than the Gram-positive bacteria. *K. pneumoniae* and *E. coli* strains were inhibited by the 30 and 50 mg/ml concentrations of the extract. The lowest MIC values at the concentrations of 10 mg/ml and 30 mg/ml were noted for the *P. aeruginosa* strains regardless of the multidrug resistance feature of the clinical strains. Clinical Gram-positive isolates were less susceptible to the antibacterial activity of seedcake extract. The MIC was 50 mg/ml for clinical staphylococci and higher for clinical enterococci. To identify the bacteriostatic or bactericidal activity of seedcake extracts, killing curves for the reference strains were determined (Figure 2). In each analyzed case, an extract concentration of 10 mg/ml was insufficient to obtain an inhibitory or killing effect against the tested strains, and the growth parameters of the bacterial cultures under such conditions were similar to those of the control. For *P. aeruginosa* ATCC 27853 cultures, the extract concentration of 30 mg/ml inhibit bacterial growth, and after 24 hours of incubation, a cfu level equal to the initial one was detected (Figure 2A). Other bacterial cultures exposed to the same extract concentration exhibited an increase in cell number of one order of magnitude for *E. coli* ATCC 11229 and *S. aureus* ATCC6538, or two orders of magnitude for *K. pneumoniae* ATCC 700603 after 24 hours of incubation (Figure 2B-D). The 30 mg/ml concentration of seedcake extract was identified as bacteriostatic for all of the reference strains used for growth curve.

**Table 4:** **Antibiotic resistance characteristics and MIC values of seedcake extracts tested on Gram**-**negative bacterial strains**

**Bacterial strains**	**Strain characteristics**	**MIC values (mg/mL)**	**Bacterial strains**	**Strain characteristics**	**MIC values (mg/mL)**
*K. pneumoniae* ATCC 700603	reference strain	30	*P. aeruginosa* ATCC 27853	reference strain	30
*K. pneumoniae* 38	MDR; ESBL+	30	*P. aeruginosa* 9/5	MDR	10
*K. pneumoniae* 36	MDR; ESBL+	50	*P. aeruginosa* 12/3	MDR	10
*K. pneumoniae* 31	MDR; ESBL+	50	*P. aeruginosa* 14/3	MDR; carbapenemase+	10
*K. pneumoniae* 44	MDR; ESBL+	30	*P. aeruginosa* 15/3	MDR	10
*K. pneumoniae* 43	MDR; ESBL+	50	*P. aeruginosa* 49/3	S	30
*K. pneumoniae* 46	S	50	*P. aeruginosa* 82/3	MDR	30
*K. pneumoniae* 37	S	30	*P. aeruginosa* 113	MDR; carbapenemase+	30
*K. pneumoniae* 35	S	50	*P. aeruginosa* 249/P	MDR	30
*K. pneumoniae* 34	S	50	*P. aeruginosa* 3	MDR	30
*K. pneumoniae* 33	S	30	*P. aeruginosa* 12	MDR	30
*E. coli* ATCC 25922	reference strain	50	*P. aeruginosa* 14	MDR	30
*E. coli* 1426	S	50	*P. aeruginosa* 18	MDR	10
*E. coli* 1419	S	30	*P. aeruginosa* 20	MDR	10
*E. coli* 1417	S	30	*P. aeruginosa* 164	MDR	30
*E. coli* 1416	S	30	*P. aeruginosa* 0013	S	30
*E. coli* 1387	S	30	*P. aeruginosa* 0038	MDR	30
*E. coli* 1378	S	30			
*E. coli* 1303	S	50			
*E. coli* 1261	S	30			
*E. coli* 1288	S	50			
*E. coli* 1273	S	50			

The MIC tests were performed by a broth microdilution method according to the European Committee on Antimicrobial Susceptibility Testing standards (EUCAST, http://www.eucast.org). was estimated by three independed biological repetition.

MDR – acquired resistance to more than 3 groups of antibiotics; ESBL + - Extended-Spectrum Beta-Lactamases, S – sensitive, no acquired resistance.

**Table 5:** **Antibiotic resistance characteristics and MIC values of seedcake extracts tested on Gram**-**positive bacterial strains**

**Bacterial strains**	**Strain characteristics**	**MIC value [mg/mL]**
*S. aureus* ATCC 29213	Reference strain	50
*S. aureus* 1	S	50
*S. aureus* 2	S	50
*S. aureus* 3	S	>50
*S. aureus* 4	S	50
*S. aureus* 5	S	50
*S. epidermidis*1MRS	MRS	30
*S. epidermidis*2MRS	MRS	50
*S. epidermidis* 3MRS	MRS	50
*S. epidermidis* 4	S	50
*S. epidermidis*5MRS	MRS	>50
*E. faecalis* 1	S	>50
*E. faecalis* 2	S	>50
*E. faecalis* 3	S	>50
*E. faecalis* 4	S	>50
*E. faecalis* 5	S	>50

The MIC tests were performed by a broth microdilution method according to the European Committee on Antimicrobial Susceptibility Testing standards (EUCAST, http://www.eucast.org). was estimated by three independed biological repetition.

S – sensitive, no acquired resistance. MRS –methicillin-resistant staphylococci. The results are the mean values ± SD (n = 3). All results are statistically significant (P < 0.05).

Gram-negative reference strains were much more susceptible to the highest concentration of extract, which reduced the bacterial count by around 3 orders of magnitude for *K. pneumoniae* to 5 for *E.coli* and 6 for *P. aeruginosa*. The *P. aeruginosa* cells appeared to be very sensitive to the bactericidal activity of seedcake extract compounds, because after 8 hours of incubation, the cfu/ml dropped from 10^6^ to 10^2^ and after one day of incubation, no living cell was detected in the culture (Figure 2A). The data suggest that the seedcake extract obtained from the transgenic flax plants is a suitable candidate for antimicrobial action with broad spectrum and partial selectivity.

### Antimicrobial effect of standard substances

In order to verify which of the identified compounds in the seedcake extract have the greatest impact on its antibacterial action, we examined the effects on the reference strain *S. aureus* ATCC 6538 of the pure compounds that had been identified in the extract. In the experiment, the concentrations of pure compounds were close to the used concentrations of seedcake extract (sum of aglicons and glicosylated form of phenolic acids) and, with a concentration 1 or 2 mg/ml of the standard substances corresponding to 30 or 50 mg/ml of the seedcake preparation, respectively. The higher concentration of 10 mg/ml was also applied. The inhibition rate (% of growth inhibition) compared to the untreated control bacteria was measured. In addition, the inhibitory effect of the seedcake preparations used in earlier experiments with clinical strains was assessed. The data suggest that *p*-coumaric and ferulic acid make a considerable contribution to the antimicrobial properties of the seedcake extract. In the case of *p*-coumaric acid, 66.22% growth inhibition was observed at a concentration equal to the sum of its free form and its glycoside derivative in the preparation of 50 mg/ml. For ferulic acid, the growth inhibition rate was 63.5% for the same concentration level. A significantly lower inhibitory effect was observed for caffeic acid (17.8%) and SDG (Table 6). It is proposed that to reach the antimicrobial effect of caffeic acid much higher concentrations need to be used than are present in the tested seedcake extracts, as indicated by the effect of the 10 mg/ml concentration. To find out if the antimicrobial effect of phenolic acids derives from the sum or synergy of their performance, simultaneous bacterial treatment of different combinations of mixed standard substances was performed. In all of the test situations, the results are significantly higher for the simultaneous effect of two, three or four substances than for a single component, and also higher than mathematical sum of the inhibitory effects observed for standard substances used in combination. Therefore, we suggest that the tested compounds exhibit a synergistic effect. The results observed for a single SDG addition and for mixtures including SDG shown that SDG has little or no influence on the antimicrobial properties of seedcake preparations. The efficacy of the seedcake extract and *p*-coumaric and ferulic acid were compared to the activity of ampicillin and gentamicin, which are respectively the inhibitors of peptidoglycan and protein synthesis (see Additional file 2: Table S3). The enterococci group turned out to be weakly susceptible to*p*-coumaric and ferulic acids regardless of the antibiotic susceptibility profiles, so their highest concentrations (>0.25and >0.80 mg/ml, respectively) had no inhibitory effect on microbial growth. Ampicillin was efficient at 0.002 mg/ml. Staphylococci exhibited a high MIC level with phenolic acids but for several strains, a similar or even lower level than for ampicillin (0.25 mg/ml versus >0.51 mg/ml). *p*-coumaric acid was active against *S.epidermidis* MRS isolates at an equal concentration to gentamicin. The *E.coli* strains are antibiotic-susceptible bacteria, and the MICs of the phenolic acids were even 100 times higher than those for ampicillin and gentamicin for most of the tested cultures. *K. pneumoniae* isolates showed similar MICs for phenolic acids versus ampicillin. The flax compounds had the strongest impact on *Pseudomonas* growth. The ampicillin inhibitory concentration was over 10 times higher than of *p*-coumaric acid and similar to ferulic acid. For gentamicin-resistant *Pseudomonas* isolates, *p*-coumaric acid was 2–5 times more effective than the drug.

**Table 6:** **The effect of pure compounds and seedcake extract on the growth of *Staphylococcus aureus *** **ATCC 29213 cultures**

**Compounds**	**1 mg/ml of each**	**2 mg/ml**	**10 mg/ml**
p-coumaric acid	43.73% ± 0.2	66.22% ± 0.62	78.31% ± 1.2
Ferulic acid	29.99% ± 0.6	63.56% ± 0,89	78.31% ± 1.2
Caffeic acid	14.74% ± 0.5	17.82% ± 0,78	83.15% ± 1.2
SDG	4.94% ± 0.9	5.32% ± 1.0	7.41% ± 0.6
p-coumaric and ferulic acid	92.59% ± 2.3	100% ± 0.91	100% ± 0.0
p-coumaric, ferulic and caffeic acids	100% ± 0.6	100% ± 0.0	100% ± 0.0
SDG and ferulic acid.	30.35% ± 1.3	63.99% ± 0.92	82.51% ± 1.6
SDG, p-coumaric , ferulic and caffeic acids	100% ± 0.0	100% ± 0.0	100% ± 0.0
Seedcake extract	83.24% ± 1.8#	100% ± 0.2##	

The presented results are the inhibition rate compared to the control (% of growth inhibition). The results are the mean values ± SD (n = 5).

#30 mg/ml of extract contains 0.86 mg, 1.45 mg and 1.14 mg of p-coumaric, ferulic and caffeic acids, respectively, and 5.5 mg SDG, which is equal to 10 mg of dry extract, ##50 mg/ml of extract.

### Potential mechanism of antimicrobial activity of seedcake extracts

**Figure 3:**
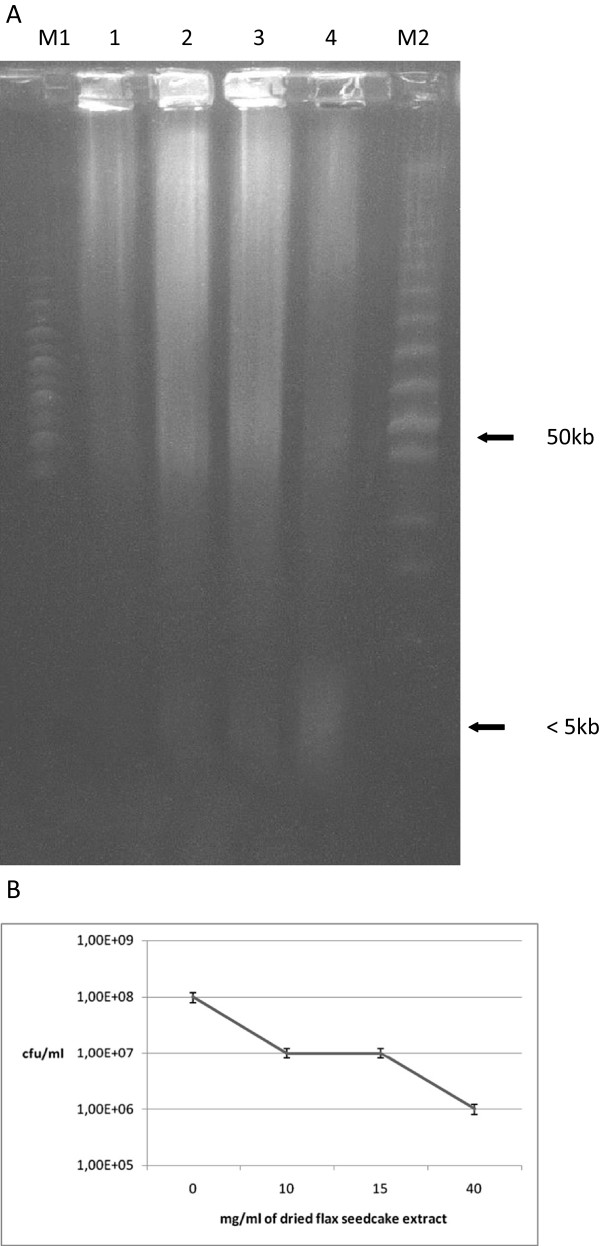
**Pulsed-field electrophoresis of seedcake treated *Escherichia coli. *A**. Line M1- MidRange Marker; line1-control (untreated bacteria); lane 2- bacteria incubated with 10 mg/ml dried seedcake extract; lane 3- bacteria incubated with 15 mg/ml dried seedcake extract; lane 4- bacteria incubated with 40 mg/ml dried seedcake extract; Lane M2- LowRange Marker. White arrow indicates ca. 50 kb, a size of typical apoptotic domain. **B**. Correlation between CFU number and seedcake extract concentration.

**Figure 4:**
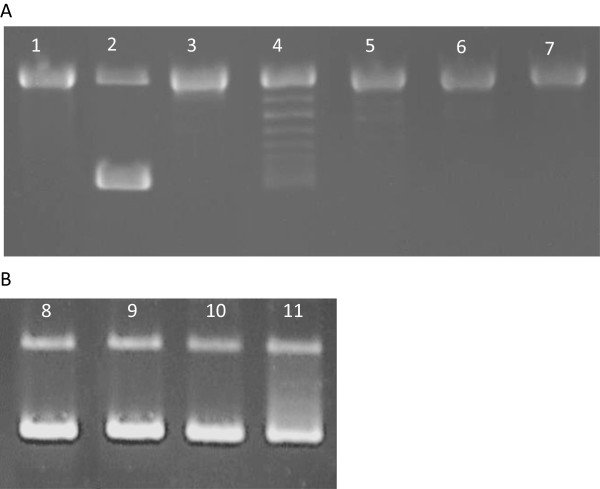
**Gyrase activity assay after treatment of seedcake extract.** Part **A**. Analysis of W92 seedcake extracts activity. Lane 1- negative control (without gyrase and extract); lane2- positive control (gyrase, without extract); lane 3- total inhibition control (novobiocin 25 μg/ml, gyrase, without extract); lane 4- reaction mixture with 10 mg/ml dried seedcake extract; lane 5- reaction mixture with 30 mg/ml dried seedcake extract; lane 6- reaction mixture with 50 mg/ml dried seedcake extract; lane 7- reaction mixture with 75 mg/ml dried seedcake extract. Part **B**. Analysis of control plant seedcake extracts activity. lane 8- positive control (gyrase, without extract), lane 9- reaction mixture with 10 mg/ml dried seedcake extract; lane 10- reaction mixture with 30 mg/ml dried seedcake extract; lane 11- reaction mixture with 50 mg/ml dried seedcake extract.

In order to investigate the mechanism of flax phenolics action on bacterial cells we analysed the integrity of bacterial genomic DNA upon cell treatment with seedcake extract by pulse-field electrophoresis (Figure 3). Results demonstrated, that bacterial DNA was visibly degraded after extract treatment but typical pattern of apoptosis (occurrence of 50 kb or 500 kb domain) was not recorded. The lower than 5 kb products of genomic DNA degradation can be observed especially after treatment with 50 mg/ml dried seedcake extract. Nucleolytic activity however remained unchanged (data not shown). The *in.vitro* test on inpact of flax seedcake extract on bacterial topoisomerase II (gyrase) activity was performed (Figure 4). Samples treated with W92 seedcake extract showed partial (10 and 30 mg/ml) or total (50 and 75 mg/ml) inhibition of gyrase activity (Figure 4A). The similar experiment using dried seedcake extract from control plants was also preformed (Figure 4B) –the slight inhibition of gyrase activity was observed only after using the highest concentration of extract (50 mg/ml). The data presented in Figures 3 and 4 suggest that phenolics cause bacteria DNA disintegration and inhibits gyrase activity. Thus to the known effect of phenolics on bacteria cell membrane we may add new targets of their action and these are gyrase activity and genomic DNA disintegration.

### Evaluation of skin irritation caused by W92 seedcake preparations

**Figure 5:**
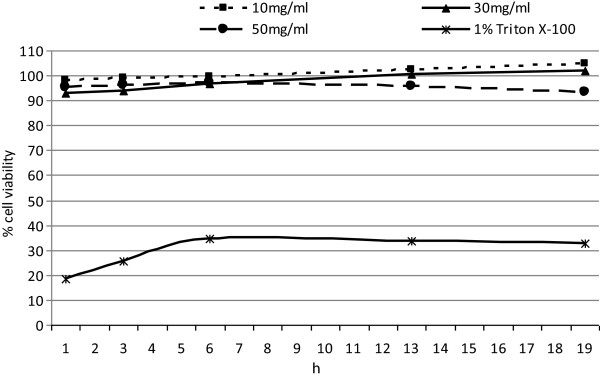
**Skin irritation potential of seedcake preparations on reconstructed Human epidermal model EpiDerm.** The irritation potential was determined for preparations 10 mg/ml, 30 mg/ml,50 mg/ml and 1% Triton X-100 as a positive control after 2, 5 and 18 h of treatment. The experiment was performed in duplicate.

To better assess the application potential of W92 seedcake preparations, the skin irritation test was performed with use of EpiDerm, a three-dimensional human skin model. This is a fully developed epidermis with a functional stratum corneum, (with keratinocytes in various stages of differentiation). It is commonly used to valuation the irritation potential of substances applied on the skin. This test can be applied as an alternative for evaluating dermal irritation prior to clinical tests. The W92 seedcake preparations were directly applied to the stratum corneum of this air-lifted, highly differentiated culture. The in vitro skin irritation evaluation was analysed 2, 5 and 18 h after application of the preparations. All analysed preparations doesn’t indicate any irritating effects on the human skin model (Figure 5). The cell viability after the application of all seedcake preparation was between 94% to 104,6% after 2, 5 and 18 h treatment. As a positive control the 1% Triton X-100 was used –this solution indicate moderate irritation effect and cell viability varied between 34,8% to 25,4% depending on time of treatment. Obtained results prove that analysed preparations appeared to be safe for dermatological applications.

## Discussion

Recently, many studies have focused on identifying potential antimicrobial agents from natural resources. The antimicrobial activity of plant extracts has been known for many years, as plants are known to produce useful antimicrobial phytochemicals [23]. Therefore, it is reasonable to try to identify a plant type that is a renewable source and provides sufficient amounts of these compounds. Plants with a certain tradition in their cultivation and with levels of antimicrobial compounds that can be increased by genetic engineering could be a sufficient source of antimicrobial preparations. Previously, a flax plant was transformed with three genes containing the cDNAs coding for key enzymes of the flavonoid biosynthesis pathway. This transgenic plant was analyzed to reveal the accumulation of flavonols and other phenolic compounds with potential antimicrobial activity. It was previously shown that the metabolite content of W92 plants, similarly to unmodified plants, varied by less than 10% from a year to year and that the stoichiometry between individual metabolites does not change either [24]. Therefore, these plants can be used as a stable source for the production of biotechnological preparations In this study, we analyzed the phenolic compound contents in extracts from seeds; seedcakes and fibers in more detail, and assessed their impact on pathogenic bacterial growth. Flavonoids are known to possess antifungal, antiviral and antibacterial activity. Moreover, several groups of flavonoids have demonstrated synergy in action within their groups and with existing chemotherapeutics [25]. It is thus hypothesized that transgenic plants overproducing flavonoids might prove to be a suitable source of antimicrobial compounds.

The seedcake extracts from the transgenic flax possess a unique composition of natural antioxidants [13]. Besides the presence of SDG, there are phenolic acids, such as ferulic, *p*-coumaric and caffeic acids and their glycosyl derivatives. Phenylpropanoids, particularly flavonoids and phenolic acids, have been found to be effective antimicrobial agents against a wide range of microorganisms. Investigations into the mechanism of action of flavonoids revealed that they have multiple cellular targets rather than one specific site of action. *E.coli* cells treated with phenolics or anthocyanins exhibited local disintegration and irregularity in their outer membrane and leakage of cytoplasm [26]. Phenolics has the unique ability to perform redox reactions at the plasma membrane and sequester electrons from the respiratory processes. The partial hydrophobicity of phenolics enables them to bind to the outer membrane and to change the membrane fluidity. The irregularity in cell shape supports the theory of localized chemical interactions at the cell surface. Once the membrane has been penetrated, smaller phenolic compounds can enter the cell and disrupt its metabolism. It is reported that the partial lipophilic nature of phenolic acids enables them to cross the cell membrane by passive diffusion in their undissociated form, and consequently disturb the cell membrane structure [27]. They cause acidification of the cytoplasm, protein denaturation, and possibly also cell death [28]. It is also suggested that pH and the length of the alkyl chain determine the antimicrobial effect of phenolic acids [28].

The data obtained strongly suggest that mainly the phenolic acids are the most effective compounds in antimicrobial activity of seedcake extract. Although the exact mechanism of this action is as yet unknown, there are several suggestions in the literature on how phenylpropanoids compounds might affect bacteria growth. It is pointed out that the mechanism of action of phenolics revealed that they have multiple cellular targets rather than one specific site of action. Phenolics bind to the outer membrane and change the membrane fluidity which might results in the local disintegration of bacterial cell membrane and cytoplasm leakage [26,27]. They might cause acidification of the cytoplasm, protein denaturation, and possibly also cell death [28].

Detailed analysis of the impact of flax seedcake extract on bacteria cell revealed new targets of extract component action and these are genomic DNA and key enzyme involved in DNA replication. Treatment of bacteria cells with seedcake extract exhibit DNA disintegration and topoisomerase II (gyrase) inhibition. It is well known that bacterial gyrase is the target of several antibiotics or over antimicrobial agents that inhibit enzyme activity [29,30]. For example novobiocin blocks the binding of ATP to gyrase while nalidixic acid interferes with the breakage and resealing of DNA chains. It is thus suggested that seedcake extract might be used as alternative antibiotic to treat multibacterial infections.

We suggest that the structural diversity within plant phenolic compounds resulted in their impact on the growth of a broad spectrum of bacterial strains. According to experiments that included an assessment of the antibacterial activity of propolis, mixtures of phenolic compounds are effective against many bacterial strains [31]. This is perhaps also the reason why the mixture of phenolic acids and lignans in GM flax extract has far more effective antibacterial activity than a pure single compound. The suggestion is further supported by the result from the experiment described in this paper where a mixture of ferulic and p-coumaric acids was evaluated. The results suggest that the activity of an individual component changed in the presence of other compounds in the extracts [32,33]. The synergy in action of extract components might explain the high antimicrobial activity of the seedcake preparation.

The analysis of influence of W92 seedcake extract on a three-dimensional human skin model (EpiDerm), which is acommonly used to assess the irritation potential of dermally applied substances, indicate no irritation effect of tested preparation. This test can be applied as an alternative for evaluating dermal irritation prior to clinical tests.

Our results show that these preparations are safe for use on the skin that we suggest that seedcake preparations might be a good candidate for treatment of bacterial infected human skin (for example acne) and an alternative to antibiotic therapy of infected wounds. The use of such preparation for human therapy (especially for injection) should be certainly preceded by clinical tests.

## Conclusion

The results of this study on genetically modified flax plants resulted in a potential new, pharmaceutical product containing biologically active compounds that exhibit antibacterial properties. Moreover, the natural products would be present in favorable proportions to counter bacterial infection according to the data. It should be pointed out that both the composition and component concentration of the product might be easily modified to give better specificity against different bacterial strains.

In this particular study, the seedcake extract was tested against sensitive and multidrug-resistant clinical bacterial strains. The obtained data are comparable to the inhibitory effect of the reference strains commonly used in antibiotic testing. The results showing the similar antibacterial activity of the flax seedcake preparation against different strains in a group of one species suggest a non-specific mode of action on bacterial cells. An interesting aspect of seedcake antibacterial activity is the bactericidal efficiency, which was highest against *P. aeruginosa* among all the tested bacteria. These bacteria are known to be the most important nosocomial pathogens that develop various mechanisms of resistance to antibiotics and antiseptic chemical compounds. It should be emphasized that these strains were isolated from severe infections where the therapy options were very limited.

The presumed mechanism of seedcake extracts action may be related to interactions between the extract component and the bacterial membrane because Gram-negative strains exhibited higher susceptibility to this preparation. Gram-positive strains were more resistant than Gram-negatives, but at higher concentrations, the seedcake extracts still inhibited the growth of staphylococci and enterococci. The results of the treatment of the drug-resistant clinical bacterial strains indicate the great application potential of seedcake flax preparations. Therefore, we suggest that flax-derived natural products are a promising alternative to antibiotic therapy. Moreover, our results show that these preparations are safe for use on the skin. Such new application possibilities of flax and especially their biomedical relevance can contribute to the renewal of flax cultivation worldwide.

## Competing interests

The authors declare that they have no competing interests.

## Authors’ contributions

MZ-carried out the biochemical analysis of seeds and seedcakes, analysis of antimicrobial effect of standard substances, drafted the manuscript, A D-J - carried out the microbiological analysis, Z D-K- interpretation of microbiological results and comparison to commercial antibiotics, MA –mechanism of antimicrobial activity analysis, AK – carried out fibre biochemical analysis. JS- conceived of the study and participate in its design and coordination. All authors read and approved the final manuscript.

## Supplementary Material

Additional file 1: Table S1 Characteristics of Gram-negative strains used. **Table S2.** Characteristic of Gram-positive strains used. Click here for file

Additional file 2: Table S3 The MIC values of seedcake extracts, standard substances and selected antibiotics tested on bacterial strains. Click here for file
