# Supplementary material for: Bactericidal activities of GM flax seedcake extract on pathogenic bacteria clinical strains
Source: BMC Biotechnol. 2014 Jul 29;14:70. doi: 10.1186/1472-6750-14-70 (PMC4134466; doi:10.1186/1472-6750-14-70)
Supplement: Additional file 1: Table S1 — Characteristics of Gram-negative strains used. Table S2. Characteristic of Gram-positive strains used. [file 1472-6750-14-70-S1.docx]

**Additional Table 1. Characteristics of Gram-negative strains used.**

R – resistant, S – sensitive AM –ampicillin; CAZ –ceftazidime; CTX –cefotaxim; CXM –cefuroxim; AK –amikacin; GM–gentamicin; CIP – ciprofloxacin; MEM – meropenem; SXT – sulfamethoxazol + trimethoprim

| Bacterial strain | Origin | Susceptibility test | | | | | | | |
| --- | --- | --- | --- | --- | --- | --- | --- | --- | --- |
|  |  | AM | CAZ | CTX | CXM | AK | GM | CIP | SXT |
| *P. aeruginosa* ATCC 27853 | American Type Culture Collection | R | S | R | R | S | S | S | R |
| *P.aeruginosa*9/5 | Nasopharynx | R | R | R | R | S | S | S | R |
| *P. aeruginosa* 12/3 | Endotracheal tube | R | S | R | R | S | S | S | R |
| *P. aeruginosa* 14/3 | Endotracheal tube | R | R | R | R | S | R | R | R |
| *P. aeruginosa* 15/3 | Nasopharynx | R | S | R | R | S | S | S | R |
| *P. aeruginosa* 49/3 | Endotracheal tube | R | S | R | R | R | S | S | R |
| *P. aeruginosa* 82/3 | Bronchoscopy fluid | R | S | R | R | S | S | S | R |
| *P. aeruginosa* 113 | Endotracheal tube | R | R | R | R | R | R | R | R |
| *P.aeruginosa 249/P* | Blood | R | S | R | R | S | S | S | R |
| *P. aeruginosa 3* | Vascular catheter | R | S | R | R | S | S | S | R |
| *P. aeruginosa 12* | Vascular catheter | R | S | R | R | S | S | S | R |
| *P. aeruginosa 14* | Vascular catheter | R | S | R | R | R | R | S | R |
| *P. aeruginosa 18* | Skin | R | S | R | R | S | S | S | R |
| *P. aeruginosa 20* | Skin | R | S | R | R | R | S | S | R |
| *P. aeruginosa 164* | Vascular catheter | R | S | R | R | S | R | S | R |
| *P.aeruginosa 0013* | Endotracheal tube | R | S | R | R | S | S | S | R |
| *P. aeruginosa 0038* | Bronchoscopy fluid | R | S | R | R | S | R | S | R |
| *K. pneumoniae* ATCC 700603 | American Type Culture Collection | R | R | R | R | R | R | S | S |
| *K. pneumoniae 38* ESBL+ | Urine | R | R | R | R | R | R | S | R |
| *K. pneumoniae 36* ESBL+ | Urine | R | R | R | R | S | S | S | S |
| *K. pneumoniae 31* | Stool | R | S | S | S | S | S | S | S |
| *K. pneumoniae 44* ESBL+ | Urine | R | R | R | R | S | S | R | R |
| *K. pneumoniae 43* ESBL+ | Stool | R | R | R | R | R | R | S | S |
| *K. pneumoniae 46* | Stool | R | R | R | R | S | R | R | R |
| *K. pneumoniae 37* | Stool | R | S | S | S | S | S | S | S |
| *K. pneumoniae 35* | Stool | R | S | S | S | S | S | S | S |
| *K. pneumoniae 34* | Urine | R | S | S | S | S | S | S | S |
| *K. pneumoniae 33* | Stool | R | S | S | S | S | S | S | S |
| *E. coli* ATCC 25922 | American Type Culture Collection | S | S | S | S | S | S | S | S |
| *E. coli* ATCC 11229 | American Type Culture Collection | S | S | S | S | S | S | S | S |
| *E.coli 1426* | Urine | S | S | S | S | S | S | S | S |
| *E.coli 1419* | Urine | R | S | S | S | S | S | S | S |
| *E.coli 1417* | Stool | S | S | S | S | S | S | S | S |
| *E.coli 1416* | Urine | R | S | S | S | S | S | S | S |
| *E.coli 1387* | Stool | S | S | S | S | S | S | S | S |
| *E.coli 1378* | Stool | S | S | S | S | S | S | S | S |
| *E.coli 1303* | Stool | R | S | S | S | S | S | S | R |
| *E.coli 1261* | Urine | R | R | R | R | S | S | S | S |
| *E.coli 1288* | Urine | S | S | S | S | S | S | S | S |
| *E.coli 1273* | Stool | S | S | S | S | S | S | S | S |

**Additional Table 2. Characteristic of Gram-positive strains used**

R – resistant, S – sensitive P– penicillin; AM– ampicillin; OX– oxacillin; E– erythromycin; L– lincomycin; GM– gentamicin, SXT– sulfamethoxazol + trimethoprim; MRS– methicillin-resistant staphylococci.

| Bacterial strain | Origin | Susceptibility test | | | | | |
| --- | --- | --- | --- | --- | --- | --- | --- |
|  |  | P/AM | OX | E | L | GM | SXT |
| *S. aureus*  ATCC 6538 | American Type Culture Collection | S | S | S | S | S | S |
| *S. aureus*  ATCC 29213 | American Type Culture Collection | R | S | S | S | S | S |
| *S. aureus* 1 | Nasopharynx | R | S | R | R | S | S |
| *S. aureus* 2 | Skin | R | S | R | R | S | S |
| *S. aureus* 3 | Vascular catheter | R | S | S | S | S | S |
| *S. aureus* 4 | Nasopharynx | S | S | R | S | S | S |
| *S. aureus* 5 | Stool | S | S | S | S | S | S |
| *S. epidermidis* 1 MRS | Vascular catheter | R | R | S | S | R | R |
| *S. epidermidis* 2 MRS | Vascular catheter | R | R | R | S | R | R |
| *S. epidermidis* 3 MRS | Blood | R | R | R | S | R | R |
| *S. epidermidis* 4 | Nasopharynx | R | S | R | S | S | S |
| *S. epidermidis* 5 MRS | Blood | R | R | R | S | R | R |
| *E. faecalis* 1 | Stool | S | - | R | R | S | R |
| *E. faecalis* 2 | Urine | S | - | R | R | R | R |
| *E. faecalis* 3 | Stool | S | - | R | R | R | R |
| *E. faecalis* 4 | Stool | S | - | R | R | S | R |
| *E. faecalis* 5 | Stool | S | - | R | R | S | R |
