# Supplementary material for: Bactericidal activities of GM flax seedcake extract on pathogenic bacteria clinical strains
Source: BMC Biotechnol. 2014 Jul 29;14:70. doi: 10.1186/1472-6750-14-70 (PMC4134466; doi:10.1186/1472-6750-14-70)
Supplement: Additional file 2: Table S3 — The MIC values of seedcake extracts, standard substances and selected antibiotics tested on bacterial strains. [file 1472-6750-14-70-S2.docx]

**Additional Table 3.TheMIC values of seedcake extracts and selected antibiotics tested on bacterial strains.**

The results are the mean values ± SD (n = 3). The results are statistically significant (P < 0.05)*. AM –ampicillin; GM –gentamicin

| Bacterial strains | MIC value (mg/ml) | | | | |
| --- | --- | --- | --- | --- | --- |
|  | Seedcake extracts | *p*-coumaric acid | Ferulic acid | AM | GM |
| *P. aeruginosa* ATCC 27853 | 30* | 0.14* | 0.50* | >0.51* | 0.002* |
| *P.aeruginosa* 9/5 | 10* | 0.05 | 0.20* | >0.51* | 0.002* |
| *P. aeruginosa* 12/3 | 10* | 0.05* | 0.20* | >0.51* | 0.002* |
| *P. aeruginosa* 14/3 | 10* | 0.05* | 0.20* | >0.51* | 0.25* |
| *P. aeruginosa* 15/3 | 10* | 0.05 | 0.20* | >0.51* | 0.002* |
| *P. aeruginosa* 49/3 | 30* | 0.14* | 0.50* | >0.51* | 0.002* |
| *P. aeruginosa* 82/3 | 30* | 0.14* | 0.50* | >0.51* | 0.002* |
| *P. aeruginosa* 113 | 30* | 0.14* | 0.50* | >0.51* | 0.25* |
| *P. aeruginosa* 249/P | 30* | 0.14* | 0.50* | >0.51* | 0.002* |
| *P. aeruginosa* 12 | 30* | 0.14* | 0.50* | >0.51* | 0.002* |
| *P. aeruginosa* 14 | 30* | 0.14* | 0.50* | >0.51* | 0.25* |
| *P. aeruginosa* 18 | 10* | 0.05* | 0.20* | >0.51* | 0.002* |
| *P. aeruginosa* 20 | 10* | 0.05* | 0.20* | >0.51* | 0.002* |
| *P. aeruginosa* 164 | 30* | 0.14* | 0.50* | >0.51* | 0.25* |
| *P. aeruginosa* 0013 | 30* | 0.14* | 0.50* | >0.51* | 0.002* |
| *K. pneumoniae* ATCC 700603 | 30* | 0.14* | 0.50* | >0.51* | 0.008* |
| *K. pneumoniae* 38 | 30* | 0.14* | 0.50* | >0.51* | 0.25* |
| *K. pneumoniae* 36 | 50* | 0.25* | 0.80* | >0.51* | 0.002* |
| *K. pneumoniae* 31 | 50* | 0.25* | 0.80* | 0.25* | 0.002* |
| *K. pneumoniae* 44 | 30* | 0.14* | 0.50* | >0.51* | 0.002* |
| *K. pneumoniae* 43 | 50* | 0.25* | 0.80* | >0.51* | 0.25* |
| *K. pneumoniae* 46 | 50* | 0.25* | 0.80* | >0.51* | 0.25* |
| *K. pneumoniae* 37 | 30* | 0.14* | 0.50* | 0.25* | 0.002* |
| *K. pneumoniae* 35 | 50* | 0.25* | 0.80* | 0.25* | 0.002* |
| *K. pneumoniae* 34 | 50* | 0.25* | 0.80* | 0.25* | 0.002* |
| *K. pneumoniae* 33 | 30* | 0.14* | 0.50* | 0.25* | 0.002* |
| *E. coli* ATCC 25922 | 50* | 0.25* | 0.80* | 0.004* | 0.001* |
| *E. coli* ATCC 11229 | 30* | 0.14* | 0.50* | 0.004 | 0.001* |
| *E.coli* 1426 | 50* | 0.25* | 0.80* | 0.004 | 0.001* |
| *E.coli* 1419 | 30* | 0.14* | 0.50* | 0.25* | 0.001* |
| *E.coli* 1417 | 30* | 0.14* | 0.50* | 0.004 | 0.001* |
| *E.coli* 1416 | 30* | 0.14* | 0.50* | 0.25* | 0.001* |
| *E.coli* 1387 | 30* | 0.14* | 0.50* | 0.004 | 0.001* |
| *E.coli* 1378 | 30* | 0.14* | 0.50* | 0.004 | 0.001* |
| *E.coli* 1303 | 50* | 0.25* | 0.80* | 0.25* | 0.001* |
| *E.coli* 1261 | 30* | 0.14* | 0.50* | 0.25* | 0.001* |
| *E.coli* 1288 | 50* | 0.25* | 0.80* | 0.004 | 0.001* |
| *E.coli* 1273 | 50* | 0.25* | 0.80* | 0.004 | 0.001* |
| *S. aeureus*ATCC 6538 | 30* | 0.14* | 0.50* | <0.001* | <0.001* |
| *S. aeureus*ATCC 29213 | 50* | 0.25* | 0.80* | 0.004 | <0.001* |
| *S. aeureus*1 | 50* | 0.25* | 0.80* | 0.032 | <0.001* |
| *S. aeureus*2 | 50* | 0.25* | 0.80* | 0.032 | <0.001* |
| *S. aeureus*3 | >50* | >0.25* | >0.80* | 0.032 | <0.001* |
| *S. aeureus*4 | 50* | 0.25* | 0.80* | <0.001* | <0.001* |
| *S. aeureus*5 | 50* | 0.25* | 0.80* | <0.001* | <0.001* |
| *S. epidermidis* 1 | 30* | 0.14* | 0.46 | >0.51* | 0.25* |
| *S. epidermidis* 2 | 50* | 0.25* | 0.80* | >0.51* | 0.25* |
| *S. epidermidis* 3 | 50* | 0.25* | 0.80* | >0.51* | 0.25* |
| *S. epidermidis* 4 | 50* | 0.25* | 0.80* | 0.032 | <0.001* |
| *S. epidermidis* 5 | >50* | >0.25* | >0.80* | >0.51* | 0.25* |
| *E. faecalis* 1 | >50* | >0.25* | >0.80* | 0.002* | 0.016 |
| *E. faecalis* 2 | >50* | >0.25* | >0.80* | 0.002* | >0.25* |
| *E. faecalis* 3 | >50* | >0.25* | >0.80* | 0.002 | >0.25* |
| *E. faecalis* 4 | >50* | >0.25* | >0.80* | 0.002* | 0.016* |
| *E. faecalis* 5 | >50* | >0.25* | >0.80* | 0.002 | 0.016* |
